# Induced Pluripotent Stem Cells for the Treatment of Lysosomal Storage Disorders

**DOI:** 10.1002/jimd.70064

**Published:** 2025-07-11

**Authors:** Maryann Lorino, Bei Qiu, Brian Bigger

**Affiliations:** ^1^ Centre for Regenerative Medicine Institute for Regeneration and Repair, the University of Edinburgh Edinburgh UK; ^2^ Division of Cell Matrix Biology and Regenerative Medicine, Faculty of Biology, Medicine and Health University of Manchester Manchester UK

## Abstract

Lysosomal disorders (LSDs) are a group of rare metabolic disorders, with an overall incidence of 1:4800 to 1:8000 live births. LSDs are primarily caused by dysfunctional lysosomal enzymes, which typically lead to the progressive accumulation of substrates within cellular lysosomes. As a result, patients experience a wide array of somatic symptoms such as visceromegaly, cardiopulmonary abnormalities, and respiratory and urinary infections. Additionally, over two‐thirds of LSD subtypes have a neurological component, and without treatment, patients experience neurodegeneration, cognitive decline, and life expectancies spanning infancy to adulthood. At present, there is no therapy that rescues the degenerative neuropathology of LSDs, and current developments, such as brain‐targeted enzyme replacement therapy, hematopoietic stem cell transplantation, and even gene therapy, can only prevent further neurodegeneration. However, recent advancements involving induced pluripotent stem cells (iPSCs) have demonstrated that stem cells may harbor the potential to both recapitulate the phenotype of neuropathic LSDs in vitro, as well as serve as a vector for regeneration in vivo, by replacing cells and neurons damaged by disease progression. This review reports the current state of iPSC technology in LSD research, and the pathway by which iPSCs are translated from disease modeling to serving as a regenerative therapeutic for neuropathic LSDs in the clinic.

## 1 Lysosomal Disorders

**FIGURE 1:**
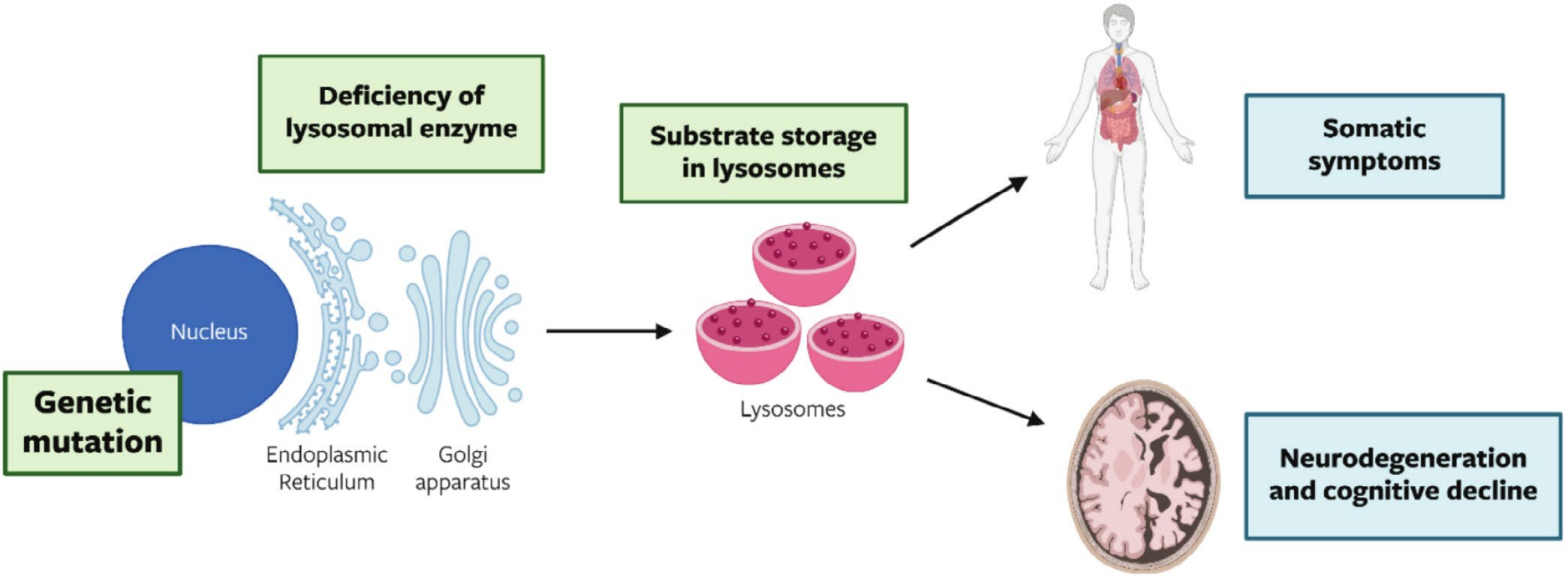
Overall pathway of lysosomal disease pathology. Specific genetic variants lead to deficiencies of mainly lysosomal enzymes, accumulation of substrates in lysosomes, and the subsequent onset of both somatic and neurological symptoms.

Lysosomal disorders (LSDs) are inborn errors of metabolism that mainly arise from defects in intrinsic lysosomal function. Others are a result of trafficking or signaling defects affecting lysosomal function. LSD patients typically lack complete or partial activity of lysosomal hydrolases, and as a result are unable to degrade complex molecules, leading to the progressive accumulation of these materials within the cell (see Figure 1). There are over 70 types of LSDs, with an overall incidence of 1:4800 to 1:8000 live births [1]. Each LSD subtype is characterized by a pathogenic variant in a single gene typically encoding for a specific lysosomal enzyme and the subsequent accumulation of undegraded complex molecules. LSDs are primarily inherited in an autosomal recessive pattern, apart from mucopolysaccharidosis (MPS) type II, Danon disease, and Fabry disease, which are X‐linked, and neuronal ceroid‐lipofuscinoses (CLN) type IV, which is autosomal dominant [1, 2].

Depending on the lysosomal defect or deficient hydrolase, as well as the complex molecule affected, the clinical manifestations of LSDs are quite variable. Overall, LSDs are progressive degenerative diseases, but patients can experience a wide range of somatic symptoms, such as visceromegaly, skeletal and cardiac dysfunction, and cardiopulmonary abnormalities, or only have specific organs affected, such as muscles or the brain (see Figure 1). Along with somatic symptoms, around two‐thirds of LSDs have some degree of neurological involvement and form the focus of this review [1].

For many LSDs, especially those with neurological symptoms, effective treatment options do not exist, and the standard care for patients is palliative, requiring a team of coordinated physicians to treat the various comorbidities of this progressive disease [3].

### 1.1 Neuropathology in LSDs

Greater than two‐thirds of the LSDs have a neurological component. While specific mechanisms underlying brain degeneration vary among LSD subtypes, neuropathic LSDs generally result in significant neuronal loss, developmental delay, intellectual disability, and patients have variable life expectancies, spanning from infancy to adulthood [1, 4]. Neurodegeneration and cognitive deficits manifest at a variety of ages, ranging from infantile to adolescence and early adulthood [5].

The neuropathic LSDs are typically divided into subgroups based on the lysosomal defect and storage substrate affected: sphingolipidoses, mucopolysaccharidoses (MPS), glycoproteinoses, and neuronal ceroid lipofuscinoses (NCL) [6].

Sphingolipidoses—including Fabry disease, Gaucher disease, Krabbe disease, metachromatic leukodystrophy (MLD), Niemann‐Pick diseases, and gangliosidosis—are characterized by pathogenic variants in enzymes that degrade sphingolipids or defects in lipid and cholesterol trafficking in the case of Niemann‐Pick disease, type C. Sphingolipids are primarily located in membranes and are critical for the structural integrity of the nervous system. As a result, patients experience symptoms affecting both the central and peripheral nervous systems. For instance, Fabry disease causes painful visceral and peripheral neuropathy, MLD involves dementia and peripheral demyelinating neuropathy, and Krabbe disease primarily causes rapid neurodegeneration in childhood [7, 8].

The MPS diseases are characterized by the storage of glycosaminoglycans (GAGs, long chain carbohydrates) in the vacuoles of lysosomes. There are 11 genetically different subtypes of MPS, each with significantly varying symptoms. For instance, MPSIII has a significant neurological phenotype, whereas MPSIV and VI patients have primarily bone and joint involvement. The MPS subtypes that possess a neuropathic phenotype are MPSI‐H (Hurler), MPSI‐HS (Hurler‐Scheie), MPSII, MPSIIIA, MPSIIIB, MPSIIIC, MPSIIID, and MPSVII. Most of these patients experience rapid cognitive decline and behavioral abnormalities, in addition to somatic symptoms such as dysmorphic facial features, corneal clouding, and recurrent respiratory and/or urinary infections [1, 9].

Glycoproteinoses, including mucolipidoses and Schindler disease, are similar to MPS diseases as patients accumulate glycoproteins within lysosomal vacuoles. Patients experience a range of neuropathic symptoms, including seizures, motor and mental regression, and ataxia [6].

NCL diseases are marked by the formation of lysosomal lipopigments in neurons and other cell types. There are 14 forms of NCL (NCL1‐NCL14), which are characterized by four key neuropathic symptoms: visual impairment, ataxia, dementia, and seizures [10].

Neuropathic LSDs outside of these categories include Pompe disease and Wolman disease, which are marked by glycogen accumulation and lipid and cholesterol accumulation, respectively. Both LSDs have severe CNS involvement, as well as harbor a myriad of somatic symptoms [1, 6].

Although this is not a comprehensive list, the common components of these diseases with disparate pathologies are progressive neurodegeneration, neuroinflammation, and loss of brain mass, including neurons. Due to the rarity of LSDs and the clinical heterogeneity of symptoms, it can be difficult for physicians to provide rapid diagnoses. In the absence of newborn screening, patients typically receive diagnoses in early childhood, after neurodegeneration and cognitive decline have begun. This highlights a critical need for early diagnostic measures, as well as treatment options that meet the needs of neurological patients who may have already experienced significant degeneration by the time of diagnosis.

## 2 Current Treatment Options

Neuropathic LSDs are particularly challenging to treat due to the presence of the blood–brain barrier (BBB) that tightly regulates and often prevents the entry of therapeutics to the CNS. To date, there are almost no effective disease‐modifying therapies available for neuropathic LSDs [11]. Current therapeutics, such as enzyme replacement therapy (ERT) and hematopoietic stem cell transplantation (HSCT), rely on rescuing enzyme expression before degeneration has begun. While these methods can offer mild improvement of somatic symptoms, they fail to deliver enough exogenous enzyme to the brain to reverse neuropathology [12, 13, 14]. The notable exception to this is HSCT treatment of the severe form of MPSI‐H, where HSCT has been the standard of care for over 20 years [9]. In addition, autologous ex vivo HSCT for presymptomatic early infantile patients with MLD has recently been approved. AAV gene therapies have also had some success at preventing neurodegeneration in presymptomatic patients with various neurodegenerative diseases, although none are yet approved for LSDs [1, 15]. Other approaches, such as small molecule therapies, only address single aspects of these multi‐faceted diseases, such as preventing the production of undegradable substrate [16, 17, 18]. As a result, these therapeutics are unable to serve as cures for LSDs but rather slow disease progression and preserve patients' cognitive state from the time of treatment.

### 2.1 Enzyme Replacement Therapy

The most common treatment for neuropathic LSDs is ERT, which aims to exogenously replace patients' deficient lysosomal hydrolase through regular intravenous infusions of the recombinant enzyme. Following administration, the enzyme is taken into cells via receptor‐mediated endocytosis through mannose‐6‐phosphate receptors and subsequently routed to the lysosome, in a process known as “cross correction” [3, 19].

While ERT has been shown to be particularly effective in non‐neuropathic LSD subtypes, alleviating many initial symptoms shown by patients, this therapy often lacks the ability to reduce primary substrate storage levels to that of an unaffected individual. Typically, ERT has only a partial effect on hard‐to‐reach organs, such as bones, joints, or cardiac tissues, and as a result, patients may suffer from an altered residual disease phenotype. In these instances, ERT has the potential to both delay and improve the disease phenotype, rather than alleviating patients of somatic symptoms completely [1, 20].

For neuropathic LSDs, standard intravenous (IV) ERT offers little neurologic benefit for patients, due to the inability of the recombinant enzyme to cross the BBB [13]. Despite this drawback, ERT remains the most widely utilized therapy for neuropathic LSDs, primarily due to its ability to improve somatic symptoms, and has been approved for MPSI, MPSII, MPSIVA, MPSVI, MPSVII, Fabry disease, Pompe disease, Gaucher disease (GD) type 1, LAL deficiency, and alpha‐mannosidosis [1, 20].

To bypass the BBB, intrathecal (IT) or intracerebroventricular (ICV) administration of recombinant enzymes enables direct delivery to the brain. ICV delivery of cerliponase‐alpha for CLN2 and idursulfase‐beta for MPSII has received approval by the FDA in the US and in Japan, respectively [1, 21, 22]. Both therapies have shown efficacy in reducing patients' neurological symptoms [1, 21, 22]. An alternative strategy involves engineering BBB‐penetrating ERTs to enhance CNS uptake following IV administration. This is typically achieved by fusing the enzyme to BBB‐crossing molecules, such as ApoB, ApoEII, FC antibody fragments, IGFII, or through nanoparticle encapsulation [1]. These approaches are under investigation for MPSI, MPSII, and MPSIIIA but have not yet received regulatory approval [1]. However, while these strategies may enhance brain targeting, neither can reverse neuronal loss that occurs early in disease onset—highlighting the likely need for regenerative therapeutic interventions.

### 2.2 Hematopoietic Stem Cell and Gene Therapies

Allogeneic HSCT is a promising strategy for a few neuropathic LSDs, such as MPSI‐H, as the progeny of transplanted hematopoietic stem cells (HSCs) can bypass the BBB to deliver functional enzyme to the brain [23]. Cross correction of LSDs via HSCT depends on the ability of HSCs to engraft and distribute as microglial cells, which can then locally provide the CNS with a permanent source of the deficient lysosomal enzyme [12].

In addition to allogeneic HSCT, ex vivo autologous HSC gene therapy involves the genetic modification of a patient's HSCs ex vivo so that they express the deficient lysosomal enzyme, without the need for immunosuppression. Atidarsagene autotemcel is the only approved HSC gene therapy for LSDs and is used for the treatment of MLD [24]. With atidarsagene autotemcel, patients' HSCs are edited to contain a functional copy of the ARSA gene so that the HSCs can produce active copies of the enzyme, arylsulfatase alpha. For symptomatic patients, this approach may have the potential to stabilize further disease progression. However, because HSCs cannot reverse neurodegeneration, HSCT is only successful at completely treating neurological disease in presymptomatic patients [25].

### 2.3 Direct Gene Therapies

Adeno‐associated viruses (AAV) are the most common in vivo gene therapy approach and aim to directly deliver missing genes to patients. AAV therapies have the capacity to reach the CNS via intracerebral, intraventricular, cerebrospinal fluid, or IV injections, by crossing the BBB [12, 19, 26]. To date, AAV has been both approved and shown to be safe and effective for treating a variety of diseases, including hemophilia and spinal muscular atrophy (SMA), but not yet for any LSD [24].

### 2.4 Substrate Reduction Therapy and Chaperone Molecules

Substrate reduction therapies (SRT) and chaperone molecules aim to inhibit the production of substrates that cannot be degraded by the lysosome and assist in the folding of mutated proteins, respectively [2, 27]. While some approved SRTs—such as miglustat for Gaucher and Niemann‐Pick disease, type C1 (NPC1) and eliglustat for GD alone—and chaperone molecules—migalastat for Fabry disease and arimoclomol for Niemann‐Pick disease, types C1 and C2—have shown benefits in delaying or improving neurological symptoms, these therapies offer little value in later stages of disease progression, as they cannot reverse CNS damage that has already occurred [1].

### 2.5 Current Therapies Do Not Address Neurodegeneration in LSDs

While current therapeutics have taken promising steps forward for treating neuropathic LSDs, their benefits wane after the onset of neurological symptoms due to the loss of neurons that coincides with symptom onset, rendering gene or enzyme replacement methodology ineffectual [15]. Due to the typical absence of obvious symptoms at birth and a critical lack of newborn screening programmes, many patients do not receive diagnoses until early childhood [1]. This presents a critical need for a novel therapeutic that addresses the neurodegenerative component of LSDs as well as meets the needs of patients with later diagnoses, who likely have experienced significant neurocognitive damage prior to treatment [28].

Recent technology involving induced pluripotent stem cells (iPSCs) may have the potential to close this therapeutic gap by serving as a vector for neural regeneration, rather than merely arresting further neurodegeneration and developmental decline. Beyond maintaining patients' quality of life, iPSC‐based therapeutics could restore the extensive CNS damage observed in neuropathic LSDs.

## 3 Induced Pluripotent Stem Cells (iPSCs)

### 3.1 Discovery and Sources of iPSCs

Before the discovery of iPSCs, human embryonic stem (ES) cells were the primary resource for stem cell research [29]. ES cells have been used to develop therapeutics for a myriad of diseases, such as Parkinson's disease and type 1 diabetes; however, due to the ethical concerns surrounding the use of human embryos, iPSCs have become a promising tool to circumvent these issues [29]. iPSCs and ES cells share many key features, such as gene expression and morphology. Additionally, both iPSCs and ES cells maintain their pluripotent character indefinitely and retain the capacity to self‐renew into any cell type in the body [29].

**FIGURE 2:**
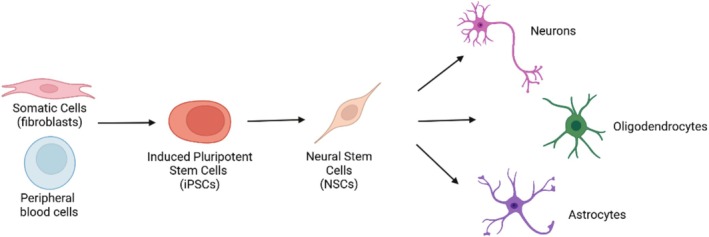
Generation of iPSCs and neural‐based products from somatic cells. First, somatic cells or peripheral blood cells are collected from human donors and subsequently developed into iPSCs. NSCs can then be generated from iPSCs and further differentiated into terminal cell types, such as neurons, oligodendrocytes, and astrocytes.

iPSCs were first established in 2006 by Yamanaka and team by using retrovirus‐mediated transduction of four essential transcription factors (c‐Myc, Oct3/4, Sox2, and Klf2) into mouse fibroblasts [29]. Following the transduction of these defined reprogramming factors, the somatic cells were reprogrammed into a pluripotent state, meaning that they regained the capacity to differentiate into cells of all three germ layers (endoderm, mesoderm, and ectoderm) [29]. By 2007, murine iPSC technology was adapted for the successful reprogramming of human fibroblasts to human iPSCs that could divide infinitely and differentiate into all cell types [30]. For instance, in the case of neural cell types, iPSCs can be differentiated into neural stem cells (NSCs), which can then give rise to multiple cell types of the nervous system, such as neurons, astrocytes, and oligodendrocytes [31] (see Figure 2). Ultimately, iPSCs can serve as an original source to generate any cell in an organism [29].

The primary sources of somatic cells for the generation of human iPSCs are dermal fibroblasts and peripheral blood cells. Because blood cells are easier to collect than the invasive skin biopsies required for fibroblast collection, peripheral blood is the most accessible resource for cellular programming and can be used to generate large quantities of cells [32].

Both unaffected individuals and patients can serve as somatic cell donors. Unaffected individuals often donate healthy somatic cells that can be transplanted to patients for allogeneic treatments. However, a critical concern regarding allogenic transplants is immunogenicity and graft versus host disease (GVHD), where the host does not accept the transplanted cells, resulting in a systemic immune response, although not all cells are immunogenic in this setting (e.g., marrow stromal cells) [33, 34]. By contrast, patient‐derived iPSCs allow for unparalleled in vitro disease modeling and the development of personal autologous therapeutics [35, 36]. For example, for the treatment of LSDs, a patient's iPSCs could be genetically modified to express the deficient enzyme and retransplanted back into the patient to restore functional enzyme activity. Additionally, patient‐derived cells retain the genetic background of individuals and eliminate any issues surrounding immune rejection from an allogeneic recipient [35, 36]. Despite these benefits, allogeneic therapies are more common than their autologous counterparts, as the same allogeneic cells can be used for multiple patients, making them more affordable and robust in application [37].

### 3.2 Genetically Modified iPSCs

Genetic editing, such as with the CRISPR/Cas9 system, can be applied to iPSC generation to create both disease‐specific and unaffected iPSC lines. First, unaffected iPSC lines can be edited to contain disease‐specific genotypes, so that they mimic patient‐specific phenotypes in vitro. These cell lines can be compared against the unedited, parent iPSC line, which serves as an isogenic control [38]. Isogenic cell lines eliminate the potential variability in phenotypes due to differences in genetic background and are an essential resource for mechanistic‐based research. Additionally, genetic editing can be applied to iPSCs derived from a diseased individual to return them to the unaffected state, which can potentially be used for in vitro modeling or autologous therapeutics.

To introduce genetic modifications, the CRISPR/Cas9 system introduces double stranded breaks (DSBs) at targeted genomic regions to subsequently activate two repair mechanisms: nonhomologous end joining (NHEJ) and homology‐directed repair (HDR). NHEJ is a simple DNA repair mechanism that allows for the deletion of large chromosomal segments in a single step, without the need for any repair template. However, because the genome can be edited multiple times by a single Cas9 enzyme, NHEJ may introduce small insertions or deletions (indels) that subsequently produce larger frameshift mutations or premature stop codons. On the other hand, HDR‐mediated gene editing is a more specific yet more complex approach that introduces single nucleotide changes or precise insertions in the genome by providing a donor DNA repair template. HDR can be used to generate iPSC lines with specific human pathogenic variants, which can more accurately recapitulate disease phenotypes in vitro. Due to the precision of these edits, cell lines generated via CRISPR/Cas9 must be screened for off‐target mutations that may be different from the anticipated genetic alteration [39, 40].

### 3.3 Methods of iPSC Generation

Understanding the method of iPSC derivation is critical in ensuring cell lines are valid for laboratory and clinical use. From their initial generation, iPSC lines must be screened for residual transcription factor activity, tumorigenic potential, and genetic mutations. Cell lines with gaps in this information should be recharacterized from the parent cell source or discarded.

As mentioned, the original approach for iPSC generation used retroviral vectors that delivered the four transcription factors to somatic cells; however, this method has become less common as the integration of constitutively expressed transgenes has the potential to disrupt the native host genome via insertional mutagenesis. It is critical that transgene expression is silenced in non‐pluripotent cell types derived from iPSCs, as residual expression can cause tumor development or disrupt downstream differentiation [41]. Lentiviruses are another retrovirus‐based method that have been adopted so that nondividing cell types, such as neurons, can be transduced with pluripotent factors; yet, because lentiviruses are integrative, this method poses the same concerns as Yamanaka's retroviral approach [32].

Methods to derive iPSCs using nonpermanent gene expression include integrating but excisable approaches, such as transposon and loxP‐flanked lentiviral systems, as well as non‐integrating methods, like plasmid or transient expression‐based systems. Excisable methods deliver the pluripotent factors as transgenes, but following successful reprogramming, the factors are excised from the host genome via self‐cleaving peptides or Cre/loxP technology [42, 43]. However, these methods require labour‐intensive screening of iPSC lines to ensure successful excision of the transgenic factors, which is a critical drawback. Another concern for the loxP‐flanked lentiviral approach is that after excision by Cre‐recombinase, loxP sites remain in the genome, generating iPSCs that are not entirely representative of the human genome [32]. Plasmid‐based approaches, such as the origin of replication/Epstein–Barr virus nuclear antigen‐1 (OriP/EBNA1) vector, deliver the pluripotent genes as a single polycistronic construct. The plasmid is delivered to host cells via nucleofection without any chromosomal integration [44]. While plasmids avoid mutagenesis caused by genome integration, they have demonstrated low reprogramming efficiency, at roughly 20%–30%, compared to other methods [45]. Additionally, plasmids can occasionally integrate into the genome, which must be monitored when generating a new iPSC line [45].

DNA‐free transgene delivery approaches are another popular choice for iPSC generation due to their inability to integrate into the host genome. The Sendai virus, the most common DNA‐free method, is a non‐pathogenic, non‐genotoxic, single‐stranded RNA virus that has high transfection efficiency in various tissues, yet lacks a DNA intermediate and thus cannot alter chromosomal DNA. Sendai viral vectors have been developed to express the four pluripotent genes. Following host cell transduction, the transgenic vector is typically expressed for 3 days, with its activity diminishing as cells are passaged, thereby minimizing residual expression [46]. However, it can be difficult to completely purge the vector from iPSC lines, and expression must be monitored when somatic cells are initially reprogrammed [32]. In addition to the Sendai virus, the expression of the pluripotent factors can be introduced to host cells via direct delivery of recombinant proteins, mRNA, or microRNA that code for each transcription factor. These methods avoid genomic integration but have demonstrated low reprogramming efficiency and a short half‐life of transduced products, necessitating further optimization before being used as a gold‐standard method [47, 48, 49].

Overall, each method of iPSC generation has its own benefits and drawbacks. Regardless of the reprogramming approach, or if genetic editing is applied, it is important to well characterize iPSCs to ensure proper expression of Yamanaka factors and confirm there is no residual expression following differentiation to downstream iPSC products [50].

### 3.4 Safety Concerns

Following iPSC generation, many safety standards must be considered before clinical translation can be achieved. The primary concern for clinical use of iPSCs is their tumorigenic potential. This fear has been raised because two pluripotent factors, c‐Myc and Klf4, are established oncogenes, and other genes involved in iPSC generation, such as Nanog, Sox2, and Oct4, have been linked to tumorgenesis [51]. Another concern surrounding tumorigenesis is associated with the expression of p53. It has been found that p53 presents a barrier to iPSC generation, as it may significantly downregulate somatic reprogramming through its intrinsic DNA damage response and by inducing apoptosis in somatic cells at the time of induction. As a result, many iPSC generation techniques, particularly those that use episomal vectors, require p53 downregulation to improve reprogramming efficiency and allow for proper expression of transgenes. However, the downregulation of p53 does not create optimal conditions for the clinic, due to its role as a tumor‐suppressor gene, emphasizing the need for further optimization of iPSC generation [52]. Finally, tumorigenesis can result from mutations acquired during iPSC generation. Both viral‐mediated and insertion‐free methods contribute to a high mutational load, as both techniques generate iPSC lines that can acquire spontaneous mutations during cell reprogramming, colony picking, and downstream culturing. Mutations acquired during or shortly after somatic reprogramming become fixed during colony expansion, which solidifies their presence in the genome. In order to generate iPSC lines free of mutations, reprogramming efficiency must be optimized to a level that no colony picking or clonal expansion is necessary [53].

### 3.5 Gold Standards for the Usage of iPSCs

To reduce concerns surrounding the safety of iPSCs, a set of recommendations and standards has been developed by the International Society for Stem Cell Research (ISSCR), which aim to ensure data reproducibility, laboratory efficacy, and a shared understanding within the field of iPSC research [50].

The first recommendation is that all cell lines should be characterized and maintained in a similar fashion, to ensure reproducibility between laboratories. Information regarding characterization and cell maintenance should be transparent for any cell line brought into the laboratory and is typically contained within a material transfer agreement (MTA), which should be readily available for all laboratory users [50].

After cell materials are obtained, a well‐characterized master cell bank should be established, so the same original source of cells can be drawn from for future experiments. Master cell banks ensure all research begins with the same, reproducible foundation, allowing for reliable data comparison. Next, cell lines should be registered with an international registry so that the line can receive a unique identity that can be recognized globally and used to track any derivatives or subclones that have been generated from the original parent line [50].

Additionally, it is critical to validate the pluripotent identity of newly generated iPSC lines. Ensuring iPSCs have the potential to differentiate into all three germ layers confirms their pluripotent identity and subsequent ability to generate progeny of multiple cell types. There are many well‐established in vitro assays for confirming differentiation potential, eliminating the need to assess pluripotency in vivo, a potential source of teratoma formation. The genomic identity of iPSCs should also be continuously monitored, as stem cells are prone to acquiring genetic changes in culture, which may affect their morphology, differentiation potential, and survival. Genomic characterization, via karyotyping or another method, of iPSCs at the initiation and termination of experiments is essential, and additional monitoring is recommended if the experimental time course exceeds 10 passages [50]. iPSCs can only be used in clinical trials after stringent selection and extensive genetic screening for mutations, in addition to the traditional pre‐clinical in vivo testing by transplanting the cells into an animalmodel [51].

In addition to in vitro iPSC usage, many standards have been adopted to ensure patient safety following translation to the clinic [50]. One critical guideline is that iPSC‐derived cell products should be delivered to patients, rather than iPSCs themselves, as iPSC products lack expression of pluripotent transgenes. For instance, for neuropathic LSDs, iPSCs can be differentiated into NSCs, purified from parental iPSCs by sorting, and then transplanted into the patient [51]. Using only well‐characterized and differentiated products reduces the chance of tumor growth and cancer development [50].

The use of good manufacturing practice (GMP) iPSC lines is also becoming an increasingly popular consideration, as proof‐of‐concept and pre‐clinical data generated from these lines can be directly translated to the clinic [50]. Because the generation of GMP lines necessitates strict regulations to ensure patient safety and clinical efficacy, many iPSC protocols in academic settings cannot easily comply with these standards, making GMP iPSC lines a particularly scarce resource. However, once generated, GMP lines can be reproduced into master banks that can be supplied to laboratories worldwide, improving the translational efficiency of iPSC research [54].

Finally, in accordance with any experimental work, accurate and detailed reporting of any research involving stem cells is critical. The ISSCR has developed a checklist of reporting practices to ensure all publications with stem cells include essential information needed for reproducibility and a universal understanding of the findings [50].

## 4 iPSCs for the Treatment and Understanding of Lysosomal Storage Disorders

In the LSD sphere, iPSC technology is rapidly becoming a resource for in vitro disease modeling and as a personalized regenerative cell therapy in the clinic. For the neuropathic LSDs, iPSCs can be differentiated into cell types of the CNS, including NSCs, neurons, and glia [31]. Recapitulating the cellular components of the CNS can help elucidate the mechanisms underpinning neurodegeneration in vitro, as well as serve as vectors for gene and cell‐based therapeutics in vivo, making iPSCs a critical tool in moving LSD research from the bench to the bedside.

### 4.1 iPSCs for LSD Disease Modeling

While animal models are most routinely used to test the in vivo efficacy of therapeutics, their inability to completely mimic the human CNS limits their usefulness for modeling neuropathic LSDs. Human iPSCs have the potential to address this gap through their capacity to differentiate into NSCs and subsequently, neurons, astrocytes, and glial cells [31]. The differentiation potential of human iPSCs can reproduce, in vitro, the different steps observed during neural development, as well as any cellular and molecular defects responsible for patients' neuropathological manifestation [55, 56]. See Table 1 for an overview of iPSC models for neuropathic LSDs that have been successfully differentiated into neural‐based products.

**TABLE 1:** Overview of iPSC models for neuropathic LSDs that have been differentiated into neural‐based products.

LSD	Reprogramming method	iPSC derivatives	Original cell source	Characterization method	Phenotype	Author
Gaucher	Retrovirus	Dopaminergic (DA) neurons	Patient fibroblasts	Karyotyping; expression of pluripotent markers; trilineage differentiation	Glucocerebrosidase (GCase) deficiency; lysosomal protein degradation; alpha‐synuclein accumulation; aggregation‐mediated neurotoxicity	Mazzulli et al. 2011 [57]
Gaucher	Lentivirus (polycistronic, Cre‐excised)	DA neurons	Patient fibroblasts	Karyotyping; expression of pluripotent markers; trilineage differentiation; teratoma formation in vivo	GCase deficiency; sphingolipid accumulation; compromised lysosomal function	Panicker et al. 2012 [58]
Gaucher	Lentivirus (polycistronic, Cre‐excised)	DA neurons	Patient fibroblasts	Karyotyping; expression of pluripotent markers; trilineage differentiation; teratoma formation in vivo	GCase deficiency	Tiscornia et al. 2013 [59]
Gaucher	Retrovirus	DA neurons	Patient fibroblasts	Karyotyping; expression of pluripotent markers; trilineage differentiation; teratoma formation in vivo	GCase deficiency; accumulation of glucosylceramide and alpha‐synuclein; autophagic and lysosomal defects; neuronal dysregulated calcium homeostasis	Schöndorf et al. 2014 [60]
Gaucher	Lentivirus (polycistronic)	NPCs, neurons	Patient fibroblasts	Karyotyping; expression of pluripotent markers; trilineage differentiation; teratoma formation in vivo	GCase deficiency; glucosylsphingosine and glucosylceramide accumulation; neuronal alpha‐synuclein accumulation; aberrant action potential firing in neurons	Sun et al. 2015 [61]
Gaucher	Preexisting lines (Panicker et al. 2012)	Neurons	Patient fibroblasts	Karyotyping; expression of pluripotent markers; trilineage differentiation; teratoma formation in vivo	GCase deficiency; lysosomal depletion; impaired autophagic flux	Awad et al. 2015 [62]
Gaucher	Lentivirus (polycistronic)	DA neurons	Patient fibroblasts	Karyotyping; expression of pluripotent markers; trilineage differentiation; teratoma formation in vivo	GCase deficiency; glucosylceramide and glucosylsphingosine accumulation; neuronal alpha‐synuclein accumulation	Aflaki et al. 2014; Aflaki et al. 2016a [63, 64]
Fabry	Sendai virus	N/A	Patient fibroblasts	Expression of pluripotent markers	Massive membranous cytoplasmic bodies in lysosomes; iPSCs were unable to be differentiated into neurons	Kawagoe et al. 2013 [65]
MLD	Retrovirus	NPCs, astrocytes	Patient fibroblasts	Karyotyping; expression of pluripotent markers; trilineage differentiation; teratoma formation in vivo	Arylsulfatase A (ARSA) deficiency	Doerr et al. 2015 [66]
MLD	Lentivirus (polycistronic, Cre‐excised)	NPCs	Patient fibroblasts	Karyotyping; expression of pluripotent markers; trilineage differentiation; teratoma formation in vivo	ARSA deficiency	Meneghini et al. 2017 [67]
MPS I	Sendai virus	NPCs	Patient fibroblasts	Karyotyping; expression of pluripotent markers	Alpha‐L‐iduronidase (IDUA) deficiency; glycosaminoglycan (GAG) accumulation; lysosomal enlargement; impaired lysosomal function	Swaroop et al. 2018 [68]
MPS II	Sendai virus	Neurons	Patient peripheral blood	Expression of pluripotent markers	Iduronate 2‐sulfatase (IDS) deficiency	Řeboun et al. 2016 [69]
MPS II	Sendai virus	Neurons, astrocytes, oligodendrocytes	Patient peripheral blood	Expression of pluripotent markers	IDS deficiency; GAG accumulation; abnormal lysosome morphology	Rybová et al. 2018 [70]
MPS II	Sendai virus	NPCs, cortical neurons	Patient peripheral blood	Karyotyping; expression of pluripotent markers; embryoid‐body formation; teratoma formation in vivo	IDS deficiency; GAG accumulation; reduced autophagic activity; neurite and axon swelling; dysregulated calcium homeostasis in neurons	Chen et al. 2024 [71]
MPS IIIB	Retrovirus	Neurons	Patient fibroblasts	Karyotyping; expression of pluripotent markers; trilineage differentiation; teratoma formation in vivo	Alpha‐N‐acetylglucosaminidase (NAGLU) deficiency; enlarged lysosomal vacuoles; enlarged Golgi apparatus; abnormalities in Golgi apparatus morphology	Lemonnier et al. 2011 [72]
MPS IIIB	Lentivirus	NPCs	Mouse embryonic fibroblasts	Expression of pluripotent markers	NAGLU deficiency	Clarke et al. 2018 [73]
MPS IIIC	Retrovirus	Neurons	Patient fibroblasts	Karyotyping; expression of pluripotent markers; trilineage differentiation; teratoma formation in vivo	Heparan‐alpha‐glucosaminidie N‐acetyltransferase (HGSNAT) deficiency; enlarged lysosomal vacuoles; impaired neuronal activity; neuronal network degradation	Canals et al. 2015 [74]
MPS VII	Retrovirus	Neurons, astrocytes	Patient fibroblasts	Karyotyping; expression of pluripotent markers; trilineage differentiation; teratoma formation in vivo	Beta‐glucuronidase deficiency	Griffin et al. 2015 [75]
MPS VII	Retrovirus	NPCs, neurons	Patient fibroblasts	Karyotyping; expression of pluripotent markers; trilineage differentiation; teratoma formation in vivo	Beta‐glucuronidase deficiency; GAG accumulation; enlarged endocytotic compartments; impaired lysosomal function; increased autophagosome number; lipid accumulation	Bayó‐Puxan et al. 2018 [76]
NCL II and III	Retrovirus	NPCs, neurons	Patient fibroblasts	Karyotyping; expression of pluripotent markers; trilineage differentiation	TPP1 and CLN3 enzyme deficiency; altered lysosome and Golgi apparatus morphology; poor yield of immature and mature neurons; neuronal substrate storage accumulation	Lojewski et al. 2014 [77]
NCL I and II	Sendai virus	NPCs	Patient fibroblasts	Karyotyping; expression of pluripotent markers	Lysosomal enlargement; lipid accumulation	Sima et al. 2018 [78]
NCL V	Sendai virus	NPCs	Patient fibroblasts	Karyotyping; expression of pluripotent markers; trilineage differentiation; embryoid‐body formation	Lipofuscin‐like material accumulation; impaired sphingolipid transport in the endo‐lysosomal pathway	Uusi‐Rauva et al. 2017 [79]
Wolman	Sendai virus	NPCs	Patient fibroblasts	Karyotyping; expression of pluripotent markers	Lysosomal acid lipase (LAL) deficiency; lipid accumulation; lysosomal enlargement	Aguisanda et al. 2017 [80]
NPA	Sendai virus	NPCs	Patient fibroblasts	Karyotyping; expression of pluripotent markers	Acid sphingomyelinase (ASM) deficiency; sphingomyelin accumulation; lysosomal enlargement	Long et al. 2016 [81]
NPC1	Lentivirus (Cre‐excised)	Neurons	Patient fibroblasts	Karyotyping; expression of pluripotent markers; trilineage differentiation; teratoma formation in vivo	Cholesterol accumulation; impaired autophagic flux; decreased cell viability	Maetzel et al. 2014 [82]
NPC1	Sendai virus	NPCs, neurons	Patient fibroblasts	Karyotyping; expression of pluripotent markers; trilineage differentiation; teratoma formation in vivo	Cholesterol accumulation	Yu et al. 2014 [83]
NPC1	Retrovirus	Neurons	Patient fibroblasts	Expression of pluripotent markers; teratoma formation in vivo	Sphingosine kinase inactivity	Lee et al. 2014 [84]
NPC1	Sendai virus	NPCs	Patient fibroblasts	Karyotyping; expression of pluripotent markers; trilineage differentiation; teratoma formation in vivo	Cholesterol accumulation; impaired autophagic flux; impaired ATP production	Soga et al. 2015 [85]
NPC1	Lentivirus	NPCs, neurons	Patient fibroblasts	Karyotyping; expression of pluripotent markers	Premature cell death, upregulation of glial cell markers, aberrant neuronal calcium and WNT signaling	Efthymiou et al. 2015 [86]
NPC1	Retrovirus	NPCs, neurons	Patient fibroblasts	Karyotyping; expression of pluripotent markers; trilineage differentiation; teratoma formation in vivo	Beta‐hexosaminidase A (Hex A) deficiency; GM2 and cholesterol accumulation	Trilck et al. 2013; Trilck et al. 2017 [87, 88]
NPC1	Retrovirus, did not go through iPSC state	NPCs	Patient fibroblasts	Expression of NPC markers	Impaired NPC self‐renewal and neuronal differentiation; cholesterol accumulation	Sung et al. 2017 [89]
GM1 gangliosidosis	Retrovirus	Neurons	Patient fibroblasts	Karyotyping; expression of pluripotent markers; trilineage differentiation; teratoma formation in vivo	Beta‐galactosidase deficiency; lysosomal enlargement; inflammasome activation; ganglioside accumulation	Son et al. 2015 [90]
GM1 gangliosidosis	Sendai virus	Neurons	Patient fibroblasts	Karyotyping; expression of pluripotent markers; trilineage differentiation; teratoma formation in vivo	GM1 ganglioside accumulation; impaired neurotransmitter release	Kajihara et al. 2020 [91]
GM2 gangliosidosis (Tay Sachs)	Sendai virus	NPCs	Patient fibroblasts	Karyotyping; expression of pluripotent markers	Lipid accumulation; lysosomal enlargement	Vu et al. 2018 [92]
GM2 gangliosidosis (Tay Sachs)	Sendai virus	NPCs, neurons	Patient fibroblasts	Karyotyping; expression of pluripotent markers; trilineage differentiation; teratoma formation in vivo	GM2 ganglioside accumulation; lysosomal enlargement; oxidative stress‐induced cell death in NPCs, neuronal reduction in exocytotic activity, impaired neurotransmitter release	Matsushita et al. 2019 [93]
GM2 gangliosidosis (Sandhoff)	Episomal vector	Cerebral organoids	Patient fibroblasts	Karyotyping; expression of pluripotent markers; trilineage differentiation	GM2 ganglioside accumulation; cerebral organoid enlargement; increased cellular proliferation; impaired neuronal differentiation	Allende et al. 2018 [94]

The sphingolipidoses are the most widely modeled LSDs. For GD, seven groups have successfully generated iPSCs from patient fibroblasts and subsequently derived neural precursor cells (NPCs) and dopaminergic neurons. Each model has replicated the disease phenotype: a lack of glucocerebrosidase activity and subsequent lysosomal sphingolipid accumulation [57, 58, 59, 60, 61, 62, 63]. Some of these models also exhibited an accumulation of alpha‐synuclein in neurons, a phenotype similar to that of Parkinson's disease, as well as impaired autophagic flux [57, 60, 61, 63]. Two models demonstrated that iPSC‐derived neurons displayed impaired signaling and dysregulated calcium homeostasis, highlighting a disruption in neuronal electrophysical properties in GD pathology [60, 61]. In modeling Fabry disease, many iPSC lines have been developed, but there has been little success in deriving neuronal cell types, highlighting a need for more robust cell lines that can model the neuropathic phenotype of this disease [65]. In the case of NPC1, an LSD characterized by impaired trafficking of cholesterol and lipids, many iPSC models have been developed from patient fibroblasts and further derived into NPCs and neurons. Widely, these models exhibit cellular cholesterol storage and impaired autophagic flux [82, 83, 84, 85, 86, 87, 89]. In addition to NPC1, one iPSC model has been developed for Niemann‐Pick disease, type A (NPA), an LSD marked by acid sphingomyelinase (ASM) deficiency [81]. The NPA iPSC model displayed a disease phenotype of ASM inactivity and sphingomyelin accumulation [81]. For MLD, an LSD characterized by deficient activity of arylsulfatase A (ARSA), two iPSC models have been developed from patient fibroblasts. Both models were derived into NPCs and lacked ARSA activity. The NPCs were transduced with lentivirus to correct ARSA expression and injected into the brains of MLD murine models to restore ARSA expression in vivo [66, 67]. Finally, to model gangliosidosis, two iPSC lines have been developed for GM1, an LSD with deficient beta‐galactosidase activity, and three for GM2, and LSD with a lack of beta‐hexosaminidase activity. The GM1 models were derived from patient fibroblasts and differentiated into NPCs and neurons [90, 91]. In addition to exhibiting defective beta‐galactosidase activity and ganglioside accumulation, one line showed that iPSC‐derived neurons displayed impaired neurotransmitter release, highlighting a potential mechanism underpinning the neurodegenerative phenotype of GM1 [91]. Similarly, the GM2 models displayed enlarged lysosomes and ganglioside accumulation [92, 93, 94]. Cerebral organoids were developed from one GM2 iPSC line, and the organoids displayed an enlarged structure due to an abnormal increase in cellular proliferation, as well as impaired neuronal differentiation [94].

For MPS, a group of LSDs marked by glycosaminoglycan (GAGs, long chain carbohydrate) accumulation, many iPSC models have been developed to investigate the disease's characteristic neurodegenerative pathology, particularly in MPSI, MPSII, MPSIIIA, MPSIIIB, MPSIIIC, and MPSVII. Regardless of the specific MPS subtype, iPSCs, NPCs, and neuron progeny exhibit deficient enzyme activity and enlarged lysosomal vacuoles [68, 69, 70, 72, 73, 74, 75, 76]. For MPSI, one iPSC model has been developed from patient fibroblasts and successfully differentiated into NPCs. This cell line lacked alpha‐iduronidase activity and demonstrated impaired lysosomal function and autophagic flux [68]. For MPSII, three iPSC lines have been developed from patients' peripheral blood, rather than from fibroblasts [69, 70, 71]. One line was differentiated into NPCs and neurons, which exhibited iduronate 2‐sulfatase (IDS) enzyme deficiency and GAG accumulation [69]. One MPSII model was differentiated into neurons, astrocytes, and oligodendrocytes and, similarly, displayed IDS enzyme deficiency and GAG accumulation [70]. Another MPSII model was differentiated into NPCs and cortical neurons, and the neurons exhibited neurite and axonal swelling, as well as dysregulated calcium homeostasis, in addition to the typical IDS deficiency and substrate storage [71]. Two iPSC models have been developed for MPSIIIB, an LSD marked by deficient N‐acetylglucosaminidase (NAGLU). One model was derived from patient fibroblasts and further differentiated into NPCs and neurons, which lacked NAGLU activity and exhibited increased lysosomal vacuole and Golgi apparatus size, a phenotype that has not been reported in other models [72]. The other model was derived from mouse embryonic fibroblasts (MEFs) and differentiated into NPCs, which also exhibited deficient NAGLU [73]. The derived murine NPCs were then transplanted into an MPSIIIB mouse model, where they appeared to activate microglia in the CNS, potentially highlighting neuroinflammatory pathways that are triggered by MPSIIIB pathology [73]. For MPSIIIC, an LSD characterized by pathogenic variants in the heparan‐alpha‐glucosaminide N‐acetyltransferase (HGSNAT) gene, one iPSC line has been developed and differentiated into NPCs and neurons. Uniquely, the derived neurons exhibited defects in electrophysical activity and network degradation, calling attention to how MPSIIIC pathology may impair the proper development of neurons during embryogenesis [74]. Finally, two iPSC models have been developed for MPSVII, a subtype defined by a beta‐glucuronidase deficiency, and differentiated into NPCs, neurons, and astrocytes [75, 76]. Both models exhibited typical disease characteristics: reduced beta‐glucosidase activity and GAG accumulation. One model exhibited enlarged endocytic compartments and an upregulation in autophagosome activity, providing insight into how MPSVII perturbs the endosomal‐lysosomal system [76].

For the NCL diseases, five iPSC models have been derived from patient fibroblasts and differentiated into NPCs [77, 78, 79]. These lines exhibited typical disease characteristics: enlarged lysosomes, reduced enzyme activity, and increased storage of lipids and lipofuscin‐like material. One model demonstrated a low yield of both immature and mature neurons during differentiation, and to date, there is no NCL iPSC line that has been successfully derived into mature neurons [77].

Finally, one model has been developed for Wolman disease, an LSD with a lysosomal acid lipase (LAL) deficiency. The iPSCs and derived NPCs exhibited key disease characteristics, such as lysosomal lipid accumulation, deficient LAL activity, and increased lysosome size [80].

In addition to iPSC models that have been differentiated into neural‐based products, a comprehensive overview of all human iPSC models generated for neuropathic LSDs has been reviewed in Borger et al. 2017 and Kido et al. 2020 [95, 96].

### 4.2 iPSCs as Regenerative Therapeutics for LSDs

Beyond simply modeling disease phenotypes in vitro, the pinnacle of iPSC technology lies in its application as a regenerative therapeutic for LSDs, addressing neural damage that has occurred prior to treatment that current therapeutics, like ERT and HSCT, cannot reverse [97]. Unlike HSCs, which are limited to generating myeloid and lymphoid lineages, iPSCs can differentiate into neurons and glial cells, offering a unique advantage in treating the neuropathic manifestations of LSDs [12]. By serving as a source of healthy neural cells, iPSCs harbor the potential to replace cells lost during disease progression, potentially restoring neurological function [31].

Additionally, iPSCs hold promise in addressing drawbacks posed by other forms of stem cell therapies. Notably, iPSCs circumvent the ethical concerns associated with ES cells, which have historically impeded research progression due to legal and regulatory constraints [98]. Furthermore, the integration of gene editing with iPSC technology enables the development of autologous, personalized therapies, minimizing the risk of immune rejection seen with allogenic ES cell or HSC treatments. Finally, as standardized guidelines—such as those provided by the ISSCR—continue to evolve and gain widespread adoption, iPSC research will expand globally, accelerating the development of regenerative therapies.

### 4.3 Murine iPSCs as a Regenerative Therapeutic

Proof‐of‐principle for the potential of stem cells to treat LSDs as a regenerative therapeutic began in 1995, when the murine‐derived immortalized neural progenitor cell line, C17.2, was transplanted into the cerebral ventricles of MPSVII mice. The NSCs were observed to successfully engraft throughout the brain, differentiate into terminal neurons, and express beta‐glucuronidase activity along the entire neuroaxis, resulting in a widespread correction of lysosomal storage in neurons and glia. However, over 94% of transplanted mice exhibited diffuse NSC engraftment, and the wild‐type level of beta‐glucuronidase expression was not sufficient to reverse neurocognitive decline. These findings highlight the need for early interventions, before the CNS is extensively damaged, as well as indicate that modifying iPSCs to express higher levels of the deficient enzyme may be necessary for a regenerative approach [99].

In this regard, in an NPA study, ex vivo gene therapy was applied to iPSCs, so that unaffected iPSC‐derived NSCs overexpressed ASM. Genetically modified NSCs were transplanted to the brains of NPA mice, which reduced lysosomal and cholesterol storage, as well as significantly increased ASM expression in the CNS, in comparison to mice transplanted with unmodified NSCs [100]. Similarly, in an early study on Krabbe disease, an LSD subtype marked by a beta‐galactocerebrosidase deficiency, transplanted murine NSCs into the Twitcher mouse model of Krabbe disease. While the engrafted NSCs produced and secreted beta‐galactocerebrosidase, they only partially restored functional enzyme activity to 20%–25% of wild‐type levels. However, when the NSCs were genetically modified to overexpress beta‐galactocerebrosidase, they demonstrated a more efficient cross correction of surrounding neurons, as well as delayed the onset of behavioral deficits and increased the lifespan of the mice [101].

The findings from these studies were critical for laying the groundwork for iPSC‐based therapeutics, and it was determined that a supraphysiological level of enzyme expression is likely necessary for iPSC‐based products to delay or reverse the neuropathology of LSDs [100, 101]. Yet, despite positive outcomes, murine iPSC‐NSC studies are not sufficient for evaluating clinical efficacy, as animal cell models cannot fully replicate the complete human disease phenotype or potential toxicities that may arise from stem cell therapies. As a result, these shortcomings have been addressed with human iPSC‐based products, which have the potential to provide a complete picture of LSD pathology in vitro and in vivo.

### 4.4 Human iPSCs as a Regenerative Therapeutic

To address gaps presented in murine iPSC studies, many groups have utilized donor and patient‐derived iPSCs to evaluate the potential of human iPSC‐based products to engraft, survive, and migrate in the CNS of LSD animal models.

In a study on MPSVII, patient‐derived iPSCs were differentiated into NSCs and genetically corrected to express beta‐glucuronidase. When the corrected NSCs were transplanted into adult MPSVII mice, they proved to reduce neuroinflammation in the CNS, as measured by a reduction in the activity of microglia. However, the NSCs engrafted primarily near the injection site and failed to migrate to surrounding brain tissue. Consequently, both beta‐glucuronidase distribution and microglial pathology were only corrected in a zone localized to the graft, limiting a complete CNS rescue [75]. Finally, in a study on NPA, human NSCs were injected into the brains of an NPA mouse model. While the NSCs were found to migrate throughout the brain and reduce cholesterol storage in sites near and far from the injection site, the migration of NSCs was dramatically reduced as the time postinjection increased, indicating that their presence in the CNS was not supported over time [102]. The spatially constrained nature of correction following transplantation in vivo highlights the need for protocols that increase cell proliferation, migration, and engraftment following NSC injection into the CNS of LSD murine models.

One approach to address the challenges surrounding NSC migration and engraftment has been to combine ex vivo gene editing with iPSC transplantation, which allows for both delivery of the deficient enzyme, as well as a mechanism to repair neural degeneration. As observed in murine iPSC‐NSC studies, it has been determined that healthy donor iPSC products often do not deliver enough enzyme to correct neuropathic LSD pathology on their own [100, 101]. Ex vivo gene therapy allows for a supraphysiological level of active enzyme to be delivered to the brain and is likely necessary for a total rescue of neurodegenerative pathology [66, 67, 101, 103, 104].

In this regard, two studies on MPSVII utilized retroviral vectors to modify unaffected iPSC‐derived NSCs so that they overexpressed beta‐glucuronidase. Following transplant to the brains of MPSVII mice, the NSCs were able to differentiate into terminal neurons, express high levels of beta‐glucuronidase for many months, and reduce primary and secondary lysosomal storage across the CNS [103, 104]. Two groups explored a similar approach for the treatment of MLD. Using patient‐derived iPSCs, both groups derived NSCs, which were genetically modified to overexpress ARSA and subsequently transplanted into an immunodeficient MLD mouse model. The transplanted NSCs successfully integrated into various brain regions, decreased sulfatide storage, and increased enzyme activity in the CNS to that of 70% of WT levels. However, the yield of NSC engraftment was highly variable and lysosomal storage was only reduced proximal to the initial injection site, highlighting gaps in iPSC engraftment that need to be addressed [66, 67]. These studies indicate that combining ex vivo gene editing with iPSC delivery may be the solution for poor stem cell engraftment, as the high enzyme level can compensate for inefficient cell survival and migration. However, further optimization is still needed to reach complete integration of transplanted stem cells and a total CNS rescue.

Aside from engraftment efficiency and enzyme delivery, a major yet unavoidable limitation surrounding xenogeneic models is the requirement of an immunosuppressive treatment prior to transplantation. In order to assess the therapeutic efficacy of human iPSC‐based products, an immunocompromised nonhuman animal model is necessary to avoid host rejection [103]. Several murine models that harbor LSD pathogenic variants have been crossed with immunodeficient murine strains to generate models that both exhibit LSD phenotypes and can accept human iPSC products, such as the NOD/SCID/GammaC chain null (NSG) X MPSIIIA mouse [105]. However, because many LSDs are accompanied by a neuroinflammatory phenotype, the immunodeficient mouse model cannot fully reflect the immune conditions present in LSD patients. These limitations must be taken into consideration when evaluating the therapeutic potential of iPSCs for the treatment of neuropathic LSDs, as inflammatory comorbidities are often a critical driver of neuropathology.

**FIGURE 3:**
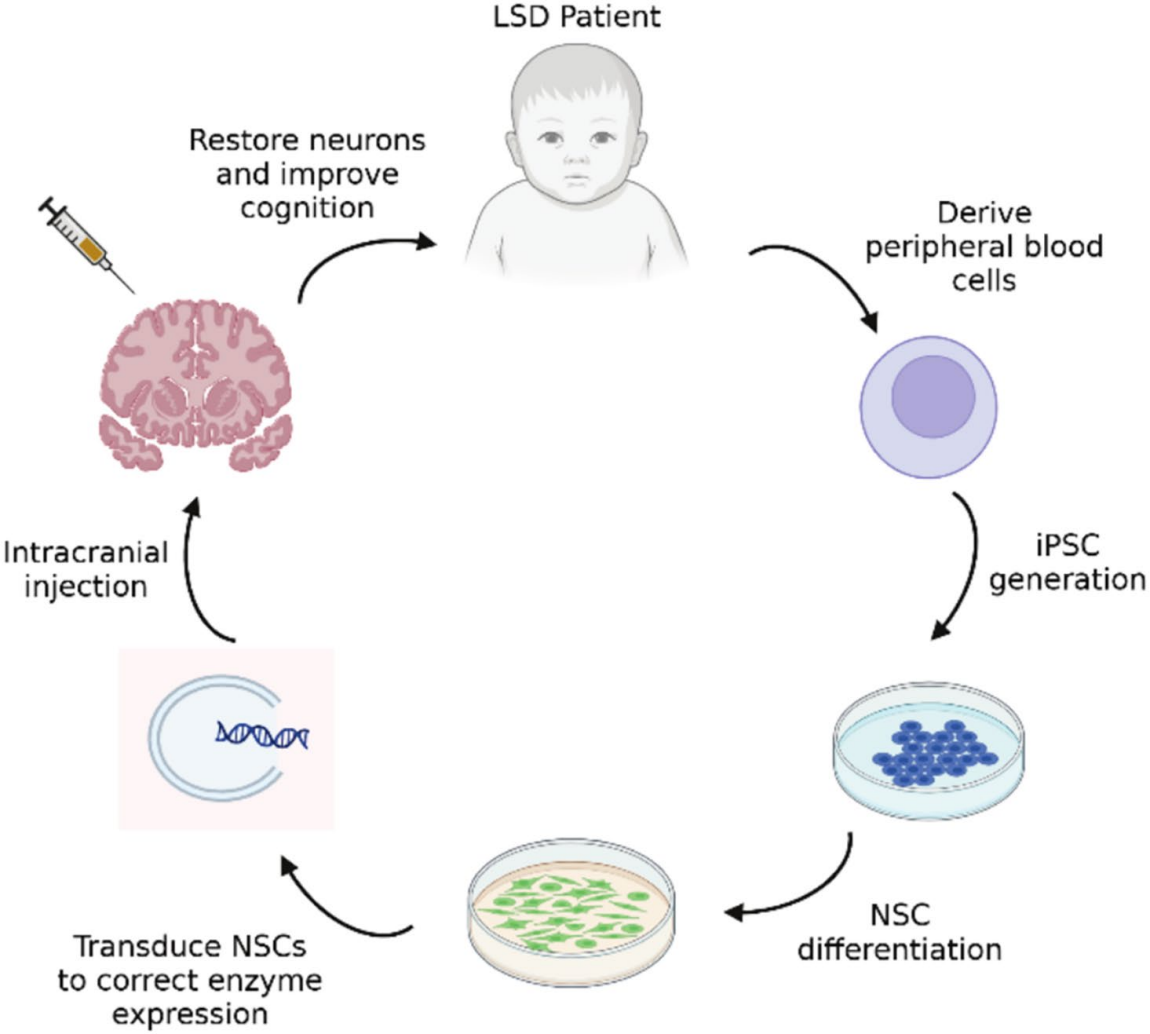
Regenerative potential of iPSCs. iPSCs and iPSC‐derived NSCs could be developed from LSD patient peripheral blood cells, genetically corrected to improve enzyme activity, and retransplanted into patients' brains to improve cognitive function.

Ultimately, while there are gaps that remain to be addressed for iPSC therapeutics, there has been substantial progress in using this cell tool as a regenerative therapeutic. The hope is that iPSC research will move toward clinical application where patients can be the donors for their own therapeutics. If successful, iPSCs derived from LSD patients could be genetically corrected and transplanted back into the patient's CNS to replace damaged and dead cells lost throughout the progression of LSDs (see Figure 3).

## 5 Future Directions

There is already significant iPSC use in the LSD space for disease modeling, and they are beginning to be investigated as a regenerative therapeutic. Many successful iPSC models, with neural‐based derivative cells, have been developed to mimic the phenotype of neuropathic LSDs in vitro. Moreover, both murine and human iPSC‐derived NSCs have been transplanted to murine models, with the aim of rescuing the neurodegenerative pathology of LSDs. Furthermore, iPSCs have been used to successfully model and treat other neurodegenerative diseases, such as Parkinson's disease, as well as metabolic disorders, like diabetes [106, 107]. Learning from these achievements may help the LSD research community address the following critical gaps needed to move iPSC‐based therapies to the clinic.

First, we anticipate and hope that the iPSC research community in LSDs will move toward generating master cell banks that are widely available in the public sphere. As mentioned in prior in “Gold Standards for the Usage of iPSCs,” a necessary step to accelerate iPSC research is to generate lines that are well karyotyped, sufficiently characterized, and cryopreserved into master cell banks, which could include derivative cell lines, such as NSCs. Additionally, we encourage academic groups to initiate collaborations with larger institutions, such as the National Institutes of Health (NIH) or the European Bank for Induced Pluripotent Stem Cells (EBiSC), which house core facilities that can efficiently develop well‐characterized iPSC lines, including lines that harbour genetic modifications. These master cell banks can be made accessible for laboratories across the world, eliminating the time needed to establish new cell lines, as well as improve the quality of mechanistic research and studies that necessitate multiple cell lines.

Secondly, the main hindrance to translating iPSC therapeutics to the clinic for the treatment of neurodegenerative diseases is the observed inability for iPSC products to fully engraft into the CNS of the host. We anticipate that iPSC research will soon move to developing stem cell models that completely integrate into the nervous system following transplantation. A step forward for integrative iPSC products is to culture functional neural networks in vitro that can then be transplanted to a recipient. This work is currently underway using iPSC‐derived colony morphology NSCs (iCoMoNSCs) [108]. This specialized collection of NSCs is uniquely homologous, self‐renewing, and following differentiation, forms mature synapses and electro‐physiologically active neurons [108]. If functionally active neural networks are transplanted to animal models, complete CNS integration and total regeneration of lost cells may be possible. The impact that reforming circuit connections after neurodegeneration would be transformative for restoring cognitive functions and behaviors that may have been lost throughout the progression of LSDs.

Another interesting direction may be to co‐transplant iPSC neural products with other cell types, such as microglia, oligodendrocytes, or astrocytes, to improve NSC engraftment following transplantation. For example, in vitro co‐cultures of microglia and NSCs have demonstrated that microglia increase the survival and proliferation of NSCs, in comparison to monocultures. Similarly, NSCs have been found to increase the phagocytic activity of microglia in co‐culture, which may be of benefit to the neuroinflammatory symptoms that often accompany LSDs [109]. Thus, a co‐culture model may address issues observed with NSC proliferation and migration in the CNS of LSD animal models. iPSC‐derived microglia have also been found to secrete lysosomal enzymes in vitro and in vivo. Culturing NSCs with microglia could improve enzyme delivery to the CNS, contributing to an improved reduction of storage molecules, as well as cross‐correcting neural cells with LSD pathogenic variants [110]. Combining NSCs with microglia, or another cell type, may be a critical next step in efficiently reversing or eliminating the progression of neuropathic pathology.

Finally, we encourage groups to explore combining iPSC‐based therapies with other therapeutic strategies. Delivering iPSC therapeutics in tandem with other therapies, such as ERT or gene therapy, may be the key to ensuring patients not only receive restoration of neuropathology, but also a rescue of lysosomal enzyme activity.

## 6 Conclusions

It is evident that iPSCs are a critical tool in identifying a regenerative solution for neuropathic LSDs. Firstly, disease‐specific iPSCs, either collected directly from patients or developed from healthy iPSCs, can provide new insights into the pathogenesis of LSDs by recapitulating the disease phenotype in vitro. Particularly, patient‐derived iPSCs can serve as a personal therapeutic, eliminating immunogenic rejection by the recipient. Yet, the frontier of iPSC technology remains in their potential to serve as regenerative therapeutics. Transplanting iPSCs in vivo allows for the production of a new cell source, replacing neurons that have been damaged throughout the progression of neuropathic LSDs. If current issues surrounding iPSC transplants are eliminated, particularly with establishing allogeneic engraftment and sustained enzyme delivery rate, iPSC derivatives may be able to reverse neurodegenerative pathologies. Moreover, if iPSCs are standardized through the generation of safe, master cell banks, the solution for LSD regeneration may be reached sooner than we anticipate. Finally, regenerative therapies of this kind will eliminate issues surrounding the age of diagnosis, bringing hope to an older patient population that is often neglected in clinical trials. We hope that this review will stimulate further interest in the development of a clinically relevant iPSC therapy for the regenerative treatment of neuropathic LSDs.

## Author Contributions

**Maryann Lorino:** conception, writing, reviewing. **Bei Qiu:** reviewing. **Brian Bigger:** conception, writing, reviewing. All authors approve the final version of this article.

## Conflicts of Interest

B.B. has received unrestricted grants and royalties from Orchard Therapeutics (now Kyowa Kirin) for work unrelated to the content of this review.

## Data Availability

The data that support the findings of this study are available on request from the corresponding author. The data are not publicly available due to privacy or ethical restrictions.
